# Investigating the distribution of calls to a North American animal poison control call center by veterinarians and the public in space, time, and space-time

**DOI:** 10.1371/journal.pone.0279299

**Published:** 2023-02-22

**Authors:** Keana Shahin, David L. Pearl, Olaf Berke, Terri L. O’Sullivan

**Affiliations:** Department of Population Medicine, Ontario Veterinary College, University of Guelph, Guelph, Canada; Universidade Federal de Minas Gerais, BRAZIL

## Abstract

Health assessments via phone call or tele-triage have become very popular. Tele-triage in the veterinary field and North American context is available since the early 2000s. However, there is little knowledge of how caller type influences the distribution of calls. The objectives of this study were to examine the distribution of calls to the Animal Poison Control Center (APCC) by caller type in space, time, and space-time. Data regarding caller location were obtained from the APCC by American Society for the Prevention of Cruelty to Animals (ASPCA). The data were analysed using the spatial scan statistic to identify clusters of higher-than-expected proportion of veterinarian or public calls in space, time, and space-time. Statistically significant spatial clusters of increased call frequencies by veterinarians were identified in some western, midwestern, and southwestern states for each year of the study period. Furthermore, annual clusters of increased call frequencies by the general public were identified from some northeastern states. Based on yearly scans, we identified statistically significant temporal clusters of higher-than-expected public calls during Christmas/winter holidays. During space-time scans of the entire study period, we identified a statistically significant cluster of higher-than-expected proportion of veterinarian calls at the beginning of the study period in the western, central, and southeastern states followed by a significant cluster of excess public calls near the end of the study period on the northeast. Our results suggest that user patterns of the APCC vary by region and both season and calendar time.

## Introduction

Veterinary tele-triage services, such as the Animal Poison Control Center (APCC) created by the American Society for the Prevention of Cruelty to Animals (ASPCA), help address gaps in access to veterinary care. The APCC is a 24 hour emergency poison control center where callers from Canada, the United States (US), as well as US commonwealths and territories can speak with veterinary toxicologists to receive veterinary advice for a pet that has been exposed to a poisonous substance [1]. The service is available to the public as well as veterinarians [1]. We previously examined factors such as dog characteristics and toxicants, to determine predictors of caller type (public or veterinarian). While we found that calls concerning certain toxicants such as food products, veterinary medicine, and human medicine, are more likely from certain types of callers, access to veterinary care was also an influential factor [2]. We also noted that a portion of the variation in caller type was attributed to county and state effects after controlling for dog-level variables and a county-level measure of access to care (i.e., veterinarians per 100,000 population) [2]. Awareness of the APCC has grown among the public, but little work has been published about the spatial and temporal distribution of calls by caller type across the US in dogs [1].

The spatial scan statistic has been used to identify spatial, temporal, and spatio-temporal clusters of infectious and non-infectious diseases in human and animal populations [3–5]. For instance, Howard-Azzeh et al., (2022) using APCC data identified clusters in space, time, and space-time of opioids and cannabinoid poisonings in dogs that were frequently consistent with human drug use patterns. However, the differential pattern in user type (i.e., veterinarian vs. the public) has yet to be explored on a national scale using the spatial scan statistic.

Consequently, the objectives of this study were the following: 1) identify clusters of calls to the APCC from veterinarians relative to the public; and 2) assess if they reflect factors previously identified in our risk factor analyses concerning caller type [2]. By determining the differential use of the APCC in space and time, its services can be tailored to better meet regional demands and temporal trends.

## Methods

### i. Data

The ASPCA provided access to the APCC AnTOX database. This dataset included the geographic coordinates, date, and caller type (veterinarian or pet owner) for each call received during the years 2005 to 2014. We analyzed caller data from 179,724 calls; 125,818 calls were from the public and 53,906 from veterinarians (Table 1).

**Table 1:** Descriptive statistics of the calls to the APCC during the study period (2005–2014).

Caller Type	Total	Percent
Veterinarian	53,906	29.99
Public	125,818	70.01
Total	179,724	100.00

### ii. Spatial, temporal, and spatiotemporal scans

Retrospective spatial, temporal, and space-time scan tests using Bernoulli models were conducted to detect statistically significant clusters of high and low proportions of calls to the APCC from veterinarians relative to the public. Bernoulli models were used where calls from veterinarians were treated as cases and calls from the public were treated as controls. For ease of interpretation, a cluster with a relative risk (RR) >1 and RR <1 were treated as veterinary clusters and public clusters, respectively. The latitude and longitude of caller locations were provided in decimal degrees with the date of the call. Spatio-temporal, and purely spatial and temporal scans were conducted for the entire study period (2005–2014) as well as purely spatial and temporal scans of each individual year.

The maximum spatial scanning window was set to 50% of the calls, we examined a two-sided hypothesis with a significance level of α = 5% to identify high and low proportions of veterinary calls relative to calls from the public. In terms of criteria for reporting secondary clusters, restrictions were placed to ensure no spatial overlap for spatial clusters. Space-time clusters were restricted to having no centers in each other’s cluster, but we only reported secondary clusters that did not overlap in space and time.

Statistical significance was determined using 999 Monte Carlo replications for purely spatial and temporal scans. The minimum temporal cluster size was set to 1 day. Statistical significance for space-time scans used sequential Monte Carlo replications with an early termination cut-off of 50 and time was collapsed in 10-day units to deal with extreme computational times. Statistically significant spatial and spatio-temporal clusters were visualized using QGIS version 3.24.3. Due to concerns over the misapplication of the term “statistically significant” [6], we highlight that the term is being used in an exploratory sense [7]. All scan statistics were applied using SaTScan version 10.0 (Kulldorff M. and Information Management Services Inc., 2021).

## Results

### i. Descriptive statistics

**Fig 1:**
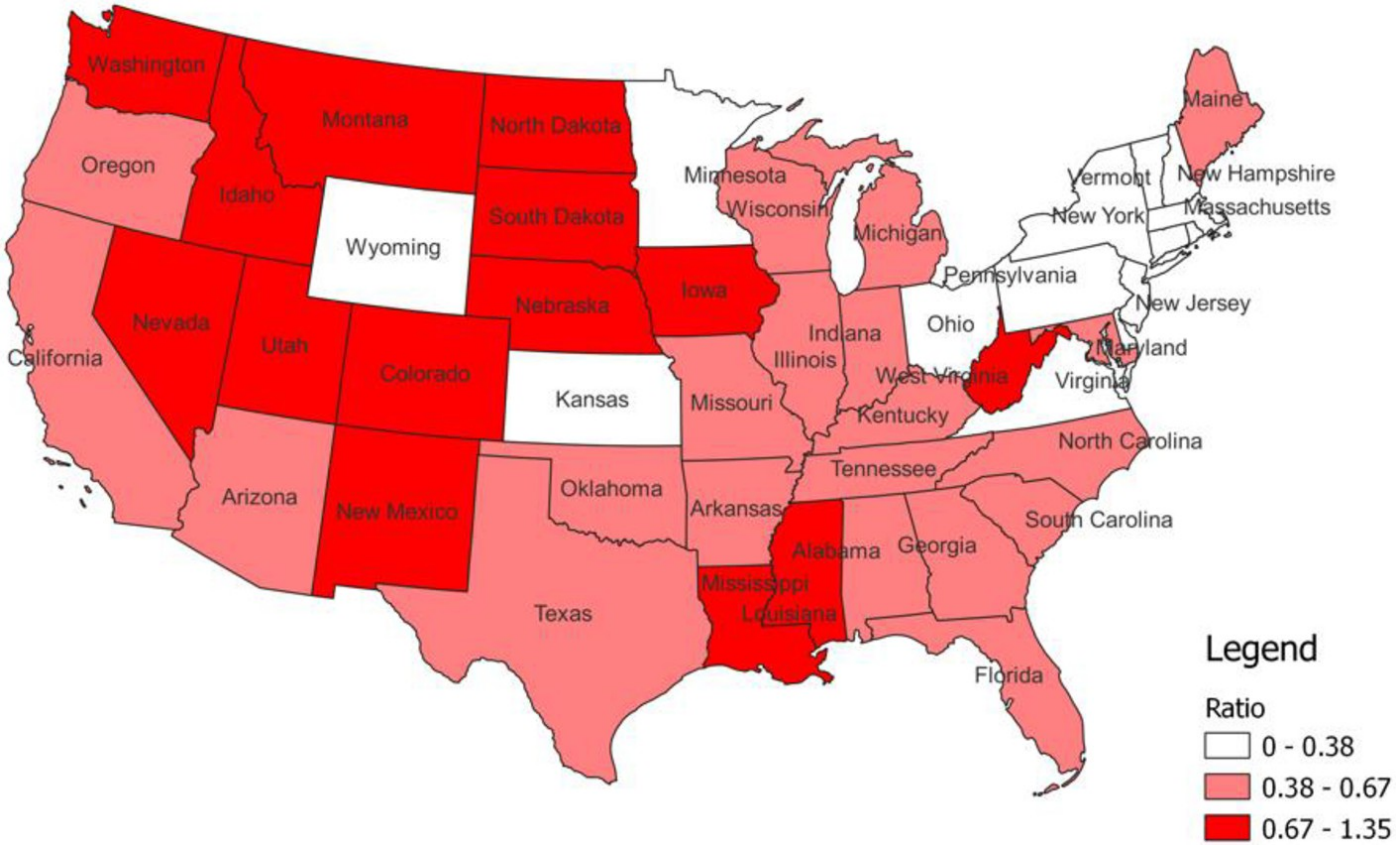
Choropleth map of the ratio of a call being from a veterinarian compared to the public in each state.

Over the study period (2005–2014), there was a greater number of calls to the APCC by the public than veterinarians (Table 1). The ratio of a call being from a veterinarian compared to the public ranged from 0 to 1.35 among US states. A number of high-ratio veterinarian states were located in midwestern, and western states, and a number of low-ratio states in the northeast (Fig 1).

### ii. Purely spatial scans

**Fig 2:**
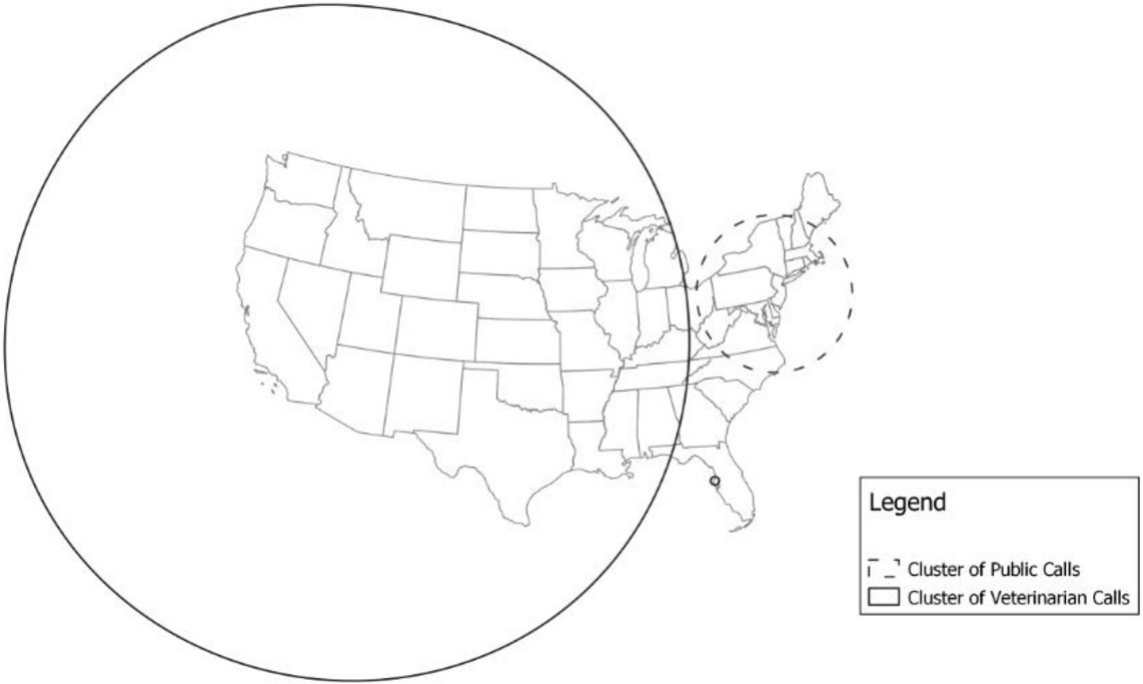
Results of the purely spatial scan of the entire study period (2005–2014), clusters with solid lines represent higher than expected veterinarian calls and dashed clusters represent higher than expected public calls.

**Fig 3:**
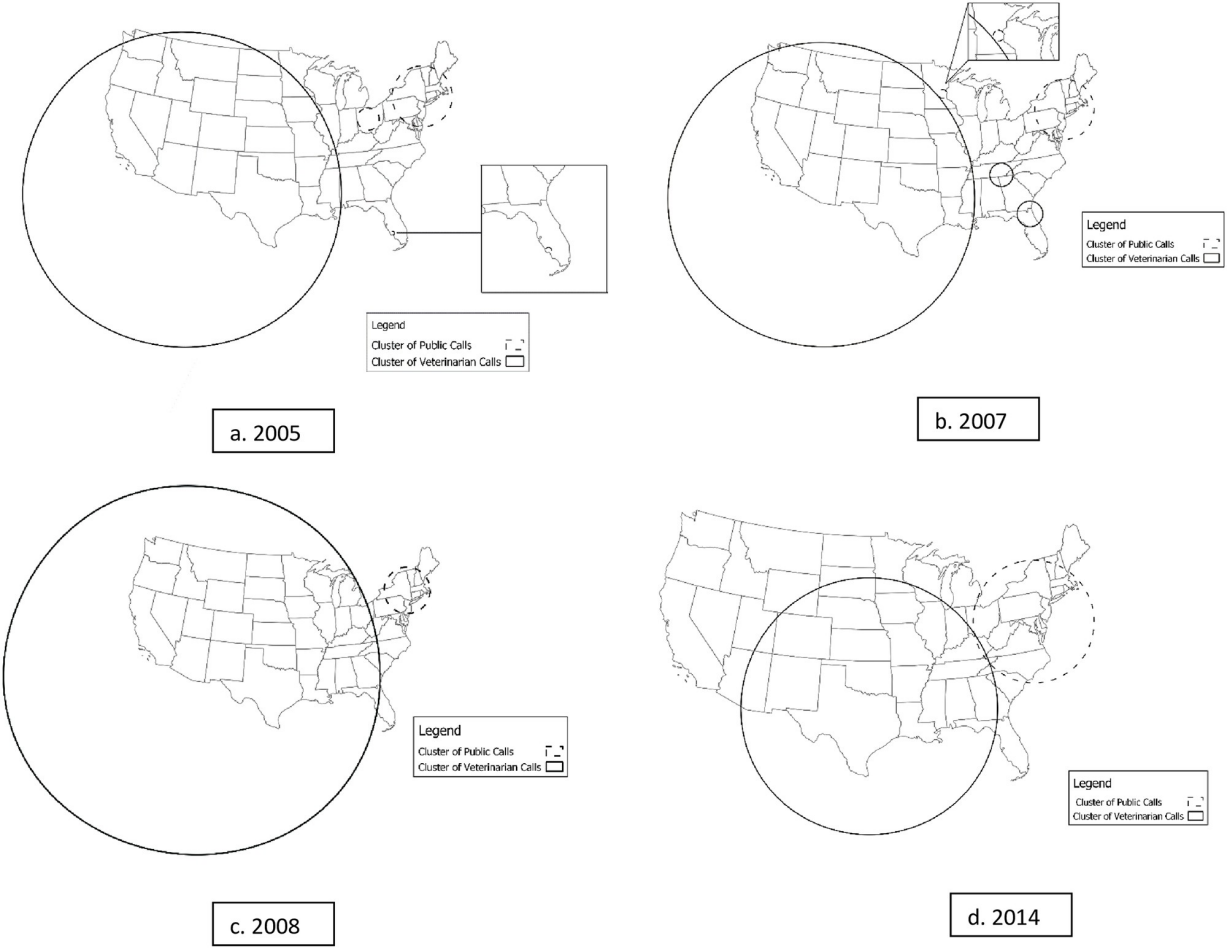
Examples of patterns identified in purely spatial scans of individual years: 2005, 2007, 2008, 2014, clusters with solid lines represent higher than expected veterinarian calls and dashed clusters represent higher than expected public calls.

Using the spatial scan test, we identified 3 statistically significant clusters (S1.05.14, S2.05.14, S3.05.14,) when examining calls during the entire study period (2005–2014), with more calls from veterinarians than expected in the west, midwestern, and southeastern states, a small cluster of higher-than-expected veterinarian calls in Florida, and more than expected calls from the public in northeastern states (Table 2, Fig 2). The purely spatial scans of individual years consistently identified a cluster of high proportion of veterinarian calls encompassing western, midwestern, and southeastern states, and a cluster with high proportion of public calls in the northeastern states (Table 2, Fig 3).

**Table 2:** Statistically significant clusters of high and low proportion of veterinarian calls relative to public calls from purely spatial scans for the entire study period (2005–2014) and each individual year*.

Year (s) Scanned	Cluster ID	Cluster Population	Number of Cases	*O*/*E*	RR	Radius (Km)	P-Value
2005–2014							
	S1.05.14	78397	17531	0.75	0.62	597	<0.001
	S2.05.14	83472	30751	1.23	1.53	2625	<0.001
	S3.05.14	739	423	1.91	1.92	32	<0.001
2005							
	S1.05	3807	1801	1.27	1.44	2085	<0.001
	S2.05	4266	1200	0.76	0.67	395	<0.001
	S3.05	211	36	0.46	0.45	144	<0.001
	S4.05	76	8	0.28	0.28	266	0.008
2006							
	S1.06	6479	1718	0.76	0.64	608	<0.001
	S2.06	3234	1565	1.39	1.55	1554	<0.001
	S3.06	74	54	2.09	2.10	135	<0.001
	S4.06	70	50	2.05	2.06	266	<0.001
2007							
	S1.07	5288	2291	1.32	1.54	2046	<0.001
	S2.07	5999	1408	0.72	0.62	397	<0.001
	S3.07	144	17	0.36	0.36	59	<0.001
	S4.07	116	66	1.74	1.75	154	0.012
	S5.07	181	94	1.59	1.60	171	0.013
2008							
	S1.08	5900	1359	0.69	0.61	343	<0.001
	S2.08	9229	3639	1.19	1.46	2801	<0.001
2009							
	S1.09	6712	1420	0.70	0.59	404	<0.001
	S2.09	9115	3385	1.22	1.57	2749	<0.001
2010							
	S1.10	9132	3302	1.24	1.63	2381	<0.001
	S2.10	8241	1784	0.74	0.62	567	<0.001
2011							
	S1.11	8494	1638	0.72	0.59	888	<0.001
	S2.11	8329	2826	1.27	1.61	2498	<0.001
	S3.11	13	12	3.46	3.47	0	0.040
2012							
	S1.12	8506	1674	0.70	0.57	578	<0.001
	S2.12	7485	2755	1.32	1.65	2337	<0.001
	S3.12	141	78	1.98	1.99	58	<0.001
2013							
	S1.13	8612	1571	0.70	0.57	573	<0.001
	S2.13	9964	3266	1.26	1.68	2701	<0.001
2014							
	S1.14	9230	1698	0.70	0.56	653	<0.001
	S2.14	4783	1622	1.29	1.42	1379	<0.001

*Each year scanned was from January 1^st^ to December 31^st^

### iii. Purely temporal scans

The purely temporal scan of the entire study period identified a single statistically significant cluster (T1.05.14), with a higher proportion of calls to veterinarians between 2005 and 2008 (Table 3). The purely temporal scans of individual years identified statistically significant clusters except in 2005 and 2006. The scans identified statistically significant temporal clusters of higher-than-expected calls from the public in December (T1.07, T1.09, T1.12, T1.13). There were statistically significant temporal clusters of higher-than-expected veterinarian calls in May 2008 (T1.08) and February 2014 (T1.14).

**Table 3:** Statistically significant clusters of high and low proportion of veterinary calls relative to public calls to the APCC from purely temporal scans of the entire study period (2005–2014) and each individual year.

Year(s) Scanned	Cluster ID	Cluster Population	Cases in Cluster	*O*/*E*	RR	P-Value	Time Frame
2005–2014	T1.05.14	62381	21437	1.15	1.24	<0.001	2005/1/5–2008/12/19
2007	T1.07	118	15	0.39	0.39	0.003	2007/12/24–2007/12/25
2008	T1.08	244	116	1.43	1.44	0.008	2008/5/19–2008/5/23
2009	T1.09	881	210	0.79	0.78	0.032	2009/12/13–2009/12/27
2011	T1.11	108	10	0.35	0.35	0.020	2011/9/24–2011/9/25
2012	T1.12	1570	363	0.83	0.81	0.016	2012/12/7–2012/12/31
2013	T1.13	446	78	0.67	0.67	0.035	2013/12/24–2013/12/29
2014	T1.14	107	49	1.74	1.75	0.041	2014/2/24–2014/2/25

*Each year scanned was from January 1^st^ to December 31^st^

### iv. Space-time scan

**Fig 4:**
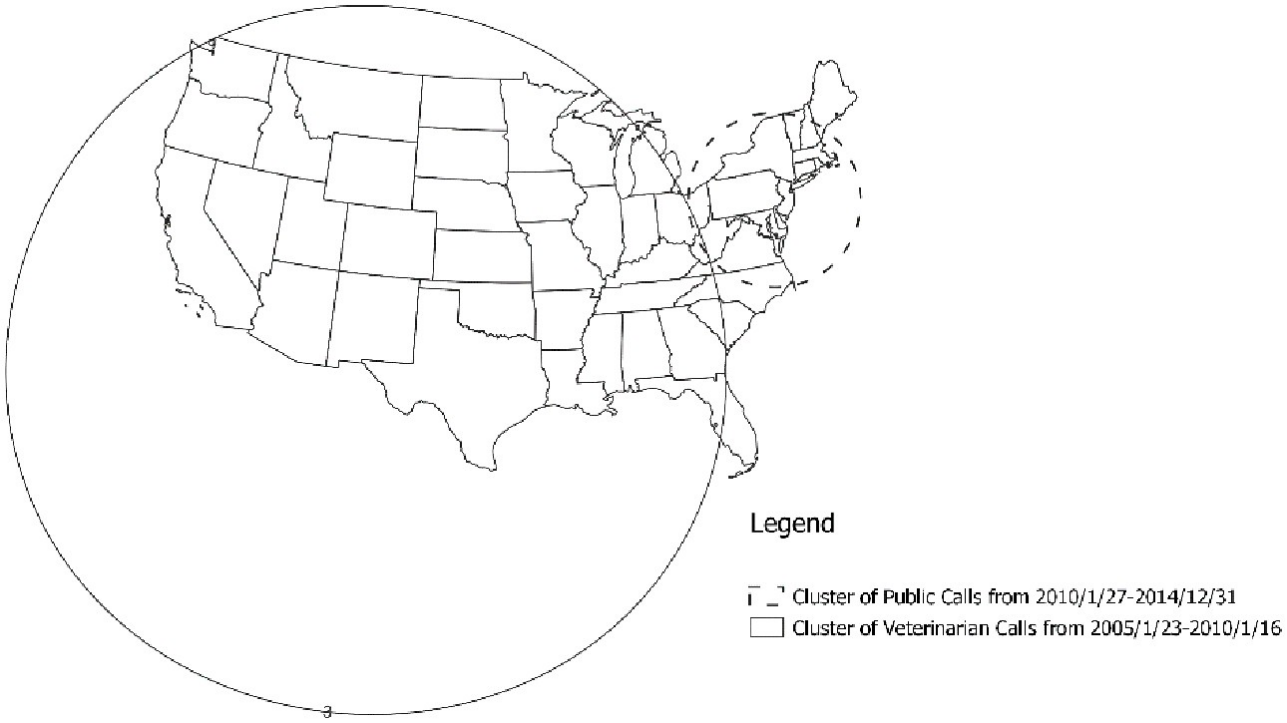
Results of the spatio-temporal scan of the entire study period (2005–2014), clusters with solid lines represent higher than expected veterinarian calls and dashed clusters represent higher than expected public calls.

The Bernoulli space-time scan of the entire study period identified a single statistically significant cluster of greater than expected calls from the public during the later half of the study period encompassing the northeastern states (ST1) and a single statistically significant cluster of higher-than-expected calls from veterinarians during the beginning of the study period encompassing the western, central, and southeastern states (ST2) (Table 4, Fig 4).

**Table 4:** Statistically significant clusters of high and low proportion of veterinary calls relative to public calls to the APCC detected from the space-time scan of the entire study period (2005–2014).

Cluster ID	Population	Number of Cases	*O*/*E*	RR	Radius (km)	Time Frame	P-value
ST1	41988	8142	0.65	0.58	569	2010/01/27-2014/12/31	<0.001
ST2	40709	16214	1.33	1.47	2367	2005/01/23-2010/01/6	<0.001

## Discussion

Past research has highlighted the utility of applying scan statistics to understand the occurrence of clusters of chronic and infectious diseases in space, time, and spacetime [3, 4, 8]. In our study, we used spatial scan statistics with a Bernoulli model, to understand the differential use of a tele-triage service between veterinarians and the general public in space, time, and space-time. In our previous work examining the differential use of the APCC by veterinarians and the public, we found regional effects impact the caller type [2]. From our previous study we noted that the odds of a call coming from a veterinarian decreased over time with the rate of decrease being slowest in areas with the greatest number of veterinarians per 100,000 population [2]. The clusters identified in space, time, and space-time were often but not always consistent with the results of our previous risk factor analysis [2].

### i. Spatial scans

The results from the spatial scans were consistent over the years with some variation in the years 2005, 2006, 2007, 2011, and 2012, where small clusters of higher-than-expected veterinarian calls were also present in southeastern states. From the individual spatial scans, we noted nearly identical clusters of higher proportions of veterinary calls in the western, midwestern, and southeastern states, and clusters with higher proportions of public calls located around northeastern states. The results of the scans were unexpected, as the clusters of higher than expected veterinarian calls for each of the years scanned included states with low numbers of veterinarians per capita [9]. From our previous work we found that some of the variance in caller type, even after accounting for characteristics and veterinarians per capita, was explained by county- and state-level factors [2]. This may indicate that there are other factors at the county- and state-level, such as socio-economic status and cultural attitudes towards pets, unrelated to those previously examined that influence caller type. In addition, based on the size of our clusters, larger regional characteristics may also play a role in who calls the APCC.

### ii. Temporal scans

Temporal scans of the entire study period identified a three-year long cluster of higher-than-expected proportion of veterinarian calls at the beginning of the study period (T1.05.14). From the scans of individual years, we identified higher-than-expected call proportions from the public during winter holiday months (T1.07, T1.09, T1.12, T1.13). This could be indicative of a seasonal effect; Weingart et al., noted a spike in food poisonings in dogs during the winter holidays and again in April [10], and Swirski et al., found that calls to the APCC for chocolate poisonings increased during Christmas and Easter holidays [1]. Our previous work also found that there was an increase in calls from the public during the winter months if the toxicant was a plant in comparison to plant calls in the fall (2). This could be due to seasonal plants (Poinsettias), or outdoor plants being brought indoors during the colder temperatures. The results from the temporal scan coupled with our previous findings indicate that there is a seasonal trend to calls to the APCC. Another possible explanation for there being an excess of public calls during the December months may be changes to veterinary clinic hours limiting access to veterinary services.

### iii. Space-time scans

Application of the space-time scan statistic identified 2 clusters: 1 of higher-than-expected proportion of calls from the public (ST1) and the other of higher-than-expected proportion of calls from veterinarians (ST2). The space-time results were consistent with the results from the purely spatial and purely temporal scans, and our previous study where the proportion of veterinary calls declined over time, but the effect varied with access to veterinarians (2). In this study, a cluster of excess veterinarian calls compared to public calls was found at the start of the study period and a cluster of excess public calls in comparison to veterinarian calls was found at the end of the study period. These findings also suggest changes in usage over time can vary by region.

### iv. Access

From this study, the spatial scans indicated that calls from veterinarians were clustered to the western, central, and southeastern states while excess calls from the public were concentrated along the northeast. However, larger geographic effects were not examined by our previous study (2); other factors which may be influencing regional effects that we did not examine could be socio-economic factors, competitive services, and cultural attitudes towards pets. For example, Blouin et al. found that dog owners in the midwest culturally view their pets as being equally important as human members of their family [11]. Owners with these attitudes may be more likely to take their dogs to the veterinarian in the event of toxicant exposure rather than call the APCC. However, Blouin et al. did not compare owner attitudes across large geographical regions so we still can only speculate on cultural factors influencing APCC usage in space and time [11].

A census of veterinarians by Salois (2019), found that states on the west coast have a high to medium concentration of veterinarians, with the midwestern states having a low to medium concentration of veterinarians [9]. The detection of clusters of higher proportions of veterinarian calls may be attributed to the number of veterinarians available in the regions. The areas where we detected spatial clusters of higher proportions of public calls to the APCC have a low to medium distribution of veterinarians (with the exception of New York State) [9]. This may be indicative of an issue of access to veterinary services, leading more pet owners to contact the APCC.

### v. Socio-economic

Some pet owners view their animals as members of the family and treat them as such, especially in terms of veterinary care [12]. Unlike human medical care, which can be paid for using government subsidized programs or work-related insurance programs, pet owners typically pay for veterinary services directly, a factor which may lead some pet owners to forego going to the veterinarian [12]. Because of this, a low-cost service like the APCC may be attractive to pet owners. However, socioeconomic status does not appear to completely determine caller type, as states known for income inequality, as measured by the Atkinson index, Gini coefficient, and Theil index, were encompassed by both clusters of higher than expected veterinarian calls (e.g., Idaho, Montana, Wisconsin, North and South Carolina) and higher than expected public calls (e.g., New York and Pennsylvania) [13]. This could be due to other factors such as awareness of the APCC, exposure to ads promoting tele-triage services, as well as availability of other veterinary services. For instance, some US humane societies offer their own 24/7 veterinary clinics.

As the field of tele-triage continues to grow, awareness of the APCC will as well, leading more pet owners to use the service. Our study has shed light on how the caller type (veterinarian or general public) to the APCC has changed over time and space, with a greater proportion of calls from pet owners using the service in recent years. While there may be extraneous factors involved in a pet owner’s decision to call the APCC or not, regional factors beyond access to veterinary care seem to be important factors in that decision and require further investigation.
